# Avian Cell Culture Models to Study Immunomodulatory Properties of Bioactive Products

**DOI:** 10.3390/ani12050670

**Published:** 2022-03-07

**Authors:** Michelle Paradowska, Aleksandra Dunislawska, Maria Siwek, Anna Slawinska

**Affiliations:** 1 Department of Animal Biotechnology and Genetics, Faculty of Animal Breeding and Biology, Bydgoszcz University of Science and Technology, Mazowiecka 28, 85-084 Bydgoszcz, Poland; aleksandra.dunislawska@pbs.edu.pl (A.D.); siwek@pbs.edu.pl (M.S.); 2 Department of Basic and Preclinical Sciences, Faculty of Biological and Veterinary Sciences, Institute of Veterinary Medicine, Nicolaus Copernicus University in Torun, Gagarina 7, 87-100 Torun, Poland; slawinska@umk.pl

**Keywords:** chicken, intestines, immunology, nutrition, organoids, prebiotics, probiotics

## Abstract

**Simple Summary:**

Bioactive products have an effect on the molecular and biochemical functions of a living organism, causing a physiological response of the given tissue. Such a products are biologically active. Depending on the active component and amount, the effects of such products can be positive or negative. Bioactive products can be food ingredients or dietary supplements, and while they are not required for survival, they are responsible for changes in the body’s health. Poultry farming struggles with zoonoses and other infectious diseases that require the use of veterinary drugs such as antibiotics. However, it is preferable to increase the natural potential of the poultry to cope with the burden of innate immune responses. Bioactive products can be used as an alternative to microbial or antiparasitic agents. Over 400,000 different plant species contain bioactive chemicals, yet only a portion of them have been examined. To examine and describe their therapeutic capabilities, more scientific analyses and characterizations are required. The use of in vitro and ex vivo models enables the evaluation of the immunomodulatory effect of bioactive molecules derived from substances such as plant extracts, essential oils, probiotics, prebiotics, and synbiotics. This article presents several studies on bioactive products and their immunomodulatory effects tested in vitro and ex vivo using various avian models.

**Abstract:**

Antimicrobial resistance is becoming a greater danger to both human and animal health, reducing the capacity to treat bacterial infections and increasing the risk of morbidity and mortality from resistant bacteria. Antimicrobial efficacy in the treatment of bacterial infections is still a major concern in both veterinary and human medicine. Antimicrobials can be replaced with bioactive products. Only a small number of plant species have been studied in respect to their bioactive compounds. More research is needed to characterize and evaluate the therapeutic properties of the plant extracts. Due to the more and more common phenomenon of antimicrobial resistance, poultry farming requires the use of natural alternatives to veterinary antibiotics that have an immunomodulatory effect. These include a variety of bioactive products, such as plant extracts, essential oils, probiotics, prebiotics, and synbiotics. This article presents several studies on bioactive products and their immunomodulatory effects tested in vitro and ex vivo using various avian cell culture models. Primary cell cultures that have been established to study the immune response in chickens include peripheral blood mononuclear cells (PBMCs), intestinal epithelial cells (IEC), and bone marrow-derived dendritic cells (BMDCs). Chicken lymphatic lines that can be used to study immune responses are mainly: chicken B cells infected with avian leukemia RAV-1 virus (DT40), macrophage-like cell line (HD11), and a spleen-derived macrophage cell line (MQ-NCSU). Ex vivo organ cultures combine in vitro and in vivo studies, as this model is based on fragments of organs or tissues grown in vitro. As such, it mimics the natural reactions of organisms, but under controlled conditions. Most ex vivo organ cultures of chickens are derived from the ileum and are used to model the interaction between the gastrointestinal tract and the microbiota. In conclusion, the use of in vitro and ex vivo models allows for numerous experimental replications in a short period, with little or no ethical constraints and limited confounding factors.

## 1. Introduction

Poultry production in Poland develops dynamically, especially in the broiler sector. Due to the large scale of the poultry production, the use of antibiotics is often required to reduce disease outbreaks [1]. Since 2009, the European Union has implemented legislation that aims to reduce the number of veterinary antibiotics in the poultry production. The use of antibiotic growth promoters in the chicken industry has been also reduced due to rising bacterial resistance to synthetic antibiotics and greater public awareness of health and food safety issues. This issue prompted researchers, the poultry industry, and the sector at large to look for safe alternatives to antibiotic growth promoters and focus on creating better long-term feed management solutions to boost chicken intestinal health and growth [2]. The trend to reduce antibiotics is correlated with the increased use of bioactive products that can have a positive effect on poultry growth, immune system, and health [3]. It is also worth noting that better animal health may lead to better food safety and quality, which benefits the consumers [4].

Bioactive products have an effort on the molecular and biochemical functions of living organisms, causing the physiological response of a given tissue. Such products are biologically active. Depending on the active component and dosage, the effects of such products can be positive or negative. Bioactive products consist of molecules that can have therapeutic effects such as reducing proinflammatory states, oxidative stress, and metabolic disorders [5]. Bioactive products can be food ingredients or dietary supplements, and while they are not required for survival, they are responsible for changes in the body’s health [6]. Sources of bioactive products are plants (herbs and spices) and certain foods (fruits, vegetables, nuts, oils, and whole grains), but are also found in living organisms and microorganisms such as bacteria or fungi [6].

Immunomodulation refers to any process that modifies the immune system or immune responses triggered by an immunomodulator [7]. Immune responses can be enhanced with immunostimulants or natural compounds, synthetic chemicals, and microorganisms that are capable of modulating the function of the immune system [8]. Many immunomodulators are synthetic or semi-synthetic, and there is growing interest in natural compounds. Consumers consider natural products with pharmacological properties and therapeutic effects as being safer than manufactured chemicals. Natural compounds originating from plants and microbes have long been recognized as useful in drug discovery and development [9].

There is a close interaction between a balanced diet and healthy guts [10]. Many studies confirm that the condition of the intestinal microbiota is directly related to the development and maintenance of the immune system in animals [11]. Early feeding with a properly selected bioactive product causes colonization of the intestines with beneficial microbiota. The interaction of microbiota with the host has positive effects on the maturation of the immune system of chicks, and induces sufficient immune responses in growing chickens [12]. The basic role of the intestines is to process food through digestion and absorption of various nutrients [13]. The supply of the nutrients to the milieu of the body is also associated with the metabolic activity of commensal or pathogenic microorganisms colonizing the intestines. The intestinal immune system must be constantly ready to react and fight pathogenic microbes, while at the same time it must be tolerant towards the commensal microorganisms inhabiting the intestines [14]. Gut-associated lymphoid tissue (GALT) comprises the intestinal part of the mucosa-associated lymphoid tissue (MALT). GALT in chickens consists of lymphoid cells and follicles, Meckel’s diverticulum, Peyer’s patches, ceacal tonsils, and bursa of Fabricius. These structures include enterocytes, goblet cells responsible for the production of the mucus, cells that produce hormones, and peptides with antimicrobial activity. Both innate and adaptive immune responses take part in GALT. The immune response is initiated with antigens sensed by respective receptors. Recognition of the antigen by the receptor triggers cellular signal transduction cascade, followed by cytokine secretion (cellular immune response), e.g., interleukin 10 (IL-10) or the transforming growth factor β (TGF-β). The goal of immune responses exerted by GALT is to neutralize pathogenic microbes while not affecting commensal organisms [15]. GALT is also the source of the largest population of B and T lymphocytes, which mediate specific immune responses and immune memory. Triggered by the contact with the antigen, B and T lymphocytes migrate to effector sites, where they develop specific immune responses of the intestinal mucosa.

Chicken is a well-established model for research on immune system and immune mechanisms. Using a chicken cell culture system as a model to study the immunomodulatory effects of bioactive molecules is more beneficial than using a live animal. The in vitro model is widely used due to the wide selection of options–primary cell cultures, cell lines, and ex vivo organ cultures including enteroids and organoids. It also allows for more repetitions of an experiment in a shorter time compared to in vivo models. In vitro models can also be used as a preliminary step for in vivo testing, which saves laboratory animals that otherwise would have to be sacrificed. Due to anatomical or physiological differences, it is very important to use species-specific cells or tissue cultures. This approach resulted in the intensive and rapid development of new in vitro models. The primary differences between avian and mammalian immune systems is the presence of the bursa of Fabricius, which in birds is a site of B cell development and the absence of encapsulated lymph nodes, which are replaced by the diffused lymphoid tissue in birds [10,11,16,17,18]. Data obtained with cell culture experiments translate better to in vivo studies when the in vitro research model reflects the complexity of the tissue. This is particularly true for the immune response, which engages a wide repertoire of lymphoid cells.

In this article, we review selected bioactive products and their immunomodulatory effects in poultry. We also present an overview of the avian cell culture models that facilitate the study of immunomodulatory effects in vitro and ex vivo. Due to the wide range of possibilities regarding both the compounds used and cell culture research models, it is worth becoming acquainted with the state of knowledge on this topic. Immunostimulation is important because healthy intestines are responsible for the better absorption of ingredients, and thus the better growth and health of individuals.

## 2. Immunomodulatory Properties of Bioactive Products

### 2.1. Plant Extracts

Plant extracts, such as thyme, oregano, or cinnamon, are widely used in poultry production [3,19]. Plant extracts supplemented in animal feed contain bioactive compounds that improve appetite, digestion, and prevent certain pathological conditions [20]. Bioactive compounds are plant chemicals, so-called phytochemicals, that have a positive effect on the various physiological functions of their consumer, including immune responses and health [21,22,23,24]. The influence of the phytogenic feed additives, such as herbs, spices, essential oils, or various mixtures on the health traits of different animal species is well documented. These products have been used as natural growth promoters in pigs and poultry [19,20,22,25]. Plant extracts (oregano, laurel, sage, anise, and citrus essential oils) have a positive effect on slaughter performance and the health of broiler chickens [26,27]. Some herbs and spices, including turmeric, cumin, black, and red peppers, nutmeg, mint, ginger, as well as chamomile or anise, also have immunostimulating properties [19]. The mechanisms of action of many phytochemicals have been established using avian cell lines and cultured lymphocytes [28]. Cinnamon has been tested for its immunomodulatory properties and was found to have antibacterial, antioxidant, and anti-cancer properties. Lee et al. (2011) stimulated chicken spleen lymphocytes with 25 mg/mL cinnamaldehyde (a component of cinnamon) and found increased cell proliferation when compared to the control [29]. Lower concentrations of cinamoldehydeneotheli caused the activation of macrophage growth or the inhibition of tumor cell growth [29]. Based on studies performed on in vitro and in vivo models, it is believed that plants such as rosemary or thyme may serve as alternatives to coccidiostatics [30]. Such testing is typically performed using *Eimeria* spp. challenged with different bioactive products, which directly indicates the coccidias’ sensitivity to a given compound. Studies have shown that the addition of plant extract from Bidens pilosa inhibited the penetration of parasites into intestinal cells in chickens [31]. Numerous studies show the ability of phytochemicals to prevent diseases or strengthen the immunity of chickens, but there is still a lack of information about the underlying mechanisms [8,14,16,22,23,32,33].

### 2.2. Prebiotics, Probiotics, and Synbiotics

Many bioactive compounds have immunomodulatory properties, and can affect the intestinal microbiota, as well as the host’s immune system. Some of them are described below. Prebiotics, probiotics, and synbiotics affect the development of a healthy intestinal microbiota and inhibit the development of intestinal pathogens [34].

Prebiotics are oligosaccharides that are not fermented by the host, but only by the intestinal microbiota. This way, they support the growth of the intestinal microbiota [35]. Commonly used prebiotics in poultry include inulin, fructooligosaccharides (FOS), galactooligosaccharides (GOS), soybeans-oligosaccharides (SOS), xylo-oligosaccharides (XOS), pyrodektrins, isomacaccharides (IMO), and lactulose [36]. Prebiotics are tested in vivo on livestock to determine their effects on gut microbiota, host immunomodulation, and the inhibition of microbial infections [36]. Bednarczyk et al. (2016) used three different prebiotics administered in different ways (injected in ovo, in water, or combined methods) to stimulate the intestinal microflora populations of broiler chickens. All of the methods and the three prebiotics increased feed intake and the feed conversion ratio compared to the control. Such results suggest a balanced consumption of energy and nutrients by healthy intestinal microflora, which is directly related to the better immune status of the animals [37].

Probiotics are “living microorganisms that, when given in the right amount, bring health benefits to the host” [38]. Supplemented in the right amount, they exert a positive effect on the microbial ecosystem of the host intestine, primarily ensuring a balance between commensal and pathogenic microbiota [39]. The use of probiotic supplements improves bowel function and protects the digestive tract from pathogenic organisms [40]. Some probiotics have shown protective properties against *Salmonella* [41]. Brisbin et al. (2010) investigated the effect of bacteria normally living in the digestive tract of chickens on the expression of cytokine genes in lymphoid cells [42]. These bacteria were *Lactobacillus acidophilus*, *Lactobacillus reuteri,* and *Lactobacillus salivarius*. Mononuclear cells isolated from cecal tonsils and the spleens of chickens were used in this experiment. The results indicated that mRNA expression of IL-1 was higher in spleen cells than in caecal tonsil cells. In the caecal tonsil cells, there were similarities and differences between the probiotic strains in inducing the mRNA levels of the tested cytokines IL-12p40, IL-10, IL-18, TGF-β4, and IFN-γ. *L. acidophilus* induced higher levels of IFN-γ, IL-12, and IL-1 expression than *L. reuteri* and *L. salivarius*, indicating that it has a greater ability to induce a putative Th1 response. *L. salivarius* inhibited the expression of proinflammatory cytokines and had the unique ability to generate TGF-β, suggesting that these bacteria may trigger an immune response. This experiment provides evidence that *Lactobacillus* spp. are capable of stimulating spleen and cecal tonsil cells in vitro [42].

Synbiotics are defined as synergistic combinations of probiotics and prebiotics that enhance the positive effects of these products administered alone. One of the functions of synbiotics is to improve the viability of microorganisms in the digestive tract [34]. Sunu et al. (2021) examined a synbiotic of garlic combined with *Lactobacillus acidophilus* for broilers [43]. After administration of the synbiotic, the intestinal morphology was assessed based on the height of the villi in the duodenum, jejunum, and ileum. The effect of the synbiotic on the immune organs was also assessed and an increased weight of the bursa of Fabricius, thymus, spleen, and caecal tonsil was found. Effects on pH, the number of *E. coli,* and the number of coliforms were also noted. Giving synbiotics T2 (T2 = basal feed + 4 mL synbiotic) reduced the coliform bacteria. The number of lactic acid bacteria was increased, which is beneficial for its host. The pH of the duodenum, jejunum, and ileum were significantly reduced by the synbiotics. The results showed that this treatment improved many aspects in broiler chickens such as performance, nutrient digestibility, blood profile, and intestinal health [43].

### 2.3. Immunomodulatory Effects of Bioactive Compounds

Plant extracts as well as prebiotics, probiotics, and synbiotics may have immunomodulatory properties. Table 1 summarises the bioactive products and compounds tested in chicken in vitro and in vivo models. Al-Kassie (2009) used thyme and cinnamon to test their effects on broiler performance and biochemical results [44]. This experiment showed that herbal extracts of vegetable oil can affect both performance and the immune system in broiler chickens. The H/L ratio was lowered, and the number of white blood cells increased using only small amounts of this supplement [44]. Other studies have used aqueous and ethanol extracts of *Ficus religiosa* to test the anti-coccidiosis effects in broiler chickens [45]. Brisbin et al. (2015) investigated the effects of three different *Lactobacillus* spp. on chicken macrophages in vitro and determined that lactobacilli stimulated the immune response of the macrophage-like cell line MQ-NCSU [46]. The immunostimulating potency of acemannan from *Aloe vera* was determined in spleen-derived macrophages collected from vaccinated chickens [47]. The effect of probiotic bacteria on immune cells has also been studied using in vivo models. An example of this type of research may be the dietary supplementation of lactobacilli in broiler chickens, which increased cytokine levels and T cell counts [46,47]. The probiotic also triggered an increase in antibody production and intestinal immune responses [48]. Jung et al. (2010) studied a combination of probiotics and fermented herbs and confirmed that the mixture increased the activity of the immune system and helped overcome *Salmonella gallinarum* infection in chicken [41]. Extracts from herbs and spices contain molecules (flavonoids and terpenoids) that inhibit the metabolism of inflammatory prostaglandins [41]. Supplementation of *Aloe secundiflora* in chickens increased antibody titres and concentrations of IL6 [49].

## 3. Avian Cell Culture Models for Testing Immunomodulatory Effects of Bioactive Products

### 3.1. Immune-Related Primary Cells Cultures

Primary cell cultures are derived directly from freshly harvested tissues and are grown under specific in vitro conditions. They are excellent research models due to their tissue origin, lack of modification, and similarity to the in vivo state. Chicken primary cell cultures are used to study the physiology and biochemistry of cells and the effects of drugs and toxic compounds [54]. One of the disadvantages of primary cells is that they have a limited lifespan. The most common types of primary cells are fibroblasts, keratinocytes, epithelial cells, and mesenchymal stem cells [55].

There are several protocols for the cultivation of primary cells of avian origin that can be used to study the immunomodulatory role of bioactive compounds (Table 2). One of these primary cell cultures is peripheral blood mononuclear cells (PBMCs). The advantage of PBMCs is that they are easy to access because they come from whole blood. In the study by Husáková et al. (2015), PBMCs isolated from chicken blood were used to study probiotics in vitro [52]. PMBC were used in one experiment to analyze the stimulation of toll-like receptor (TLR) ligands and live probiotics. This experiment enabled the analysis of TLR-mediated cytokine gene expression related to the immune response against substances listed in the table below (Table 2). The immune response was varied depending on the stimulating substance and the stimulation time [56]. PMBC obtained from ducks was used as cell models for the Duck Plague virus [57].

Intestinal epithelial cells (IEC) have one of the fastest turnovers and renewal abilities among primary cells. IEC enable the analysis of intestinal physiology [58]. IEC have also been used to evaluate the effects of selected *Lactobacillus* probiotics on the production of avian beta-defensin 9 (AvBD9), which is responsible for maintaining the homeostasis of the gastrointestinal microbiota and the gut immune system [59]. Research on intestinal epithelial cells is increasingly popular due to their association with immunity [60].

Chicken bone marrow-derived dendritic cells (BMDCs) originate from bone marrow cells collected from bones (usually femur and tibia) [61]. They are often used to study the responses of these cells to various types of *Salmonella* infections or the administration of vaccines. Biggelaar (2021) performed proteomic and RT-qPCR analyses of unstimulated cells using BMDCs and following the administration of the IBV+NDV vaccine, IBV antigen, and lipopolysaccharides produced by *E. coli* [62]. The RT-qPCR method was used to assess the mRNA expression level of the most elevated proteins in chicken immune cells. This research allowed for the identification of target proteins that might be used to evaluate the quality of inactivated in vitro poultry vaccines [62]. Research carried out by Matulova et al. (2012, 2013) concerned gene expression during *Salmonella enterica* infection [63,64]. These studies have shown the increased traction of genes responsible for inflammation or cytoskeletal regulation and cell migration in the bursa of Fabricius [63,64].

Other primary cell cultures can be determined using mononuclear cells isolated from the spleen or cecal tonsils, which are mainly made up of lymphocytes. Koenen et al. (2004) used splenic mononuclear cells to test for probiotic bacteria. He created an in vitro assay that may be used to screen lactic acid bacteria for immunomodulatory characteristics in chickens in vivo. Strains that have a good impact on spleen lymphocyte proliferation in vitro also have a positive impact on particular humoral immune responses in vivo [65]. Brisbin et al. (2010) established primary cultures of mononuclear cecal tonsil cells in coculture with probiotic bacteria to investigate inflammatory responses [42]. The results of this research are showed below in Table 2.

### 3.2. Immune-Related Cell Lines in Chicken

The cell line is established based on cells from a primary culture that multiply in vitro. Avian cell lines must meet several conditions to be useful for in vitro studies. The first condition is rapid proliferation, which ensures the high efficiency of cells for screening. The cell line should be relatively easy to maintain in vitro. A key condition is that the cell line is homogeneous and free from all kinds of impurities, such as other cell lines or mycoplasma [55]. The growing use of cell lines in poultry and other livestock species is due to their profitability compared to the maintenance of live animals. Cell lines provide an unlimited supply of research material, which makes the results easily reproducible [66].

Table 3 provides an overview of the avian cell lines associated with the immune system. DT40 is a B cell line established from bursa lymphoma caused by leukemia virus infection [67]. DT40 can be used for the screening and selection of synbiotics for chickens [68]. DT40 has also been used to study the molecular basis of immune response in chickens [69]. Dunislawska et al. (2017) analyzed the transcriptional activity of the immune responses against KHL (Keyhole limpet hemocyanin), LTA (lipoteichoic acid) from *Staphylococcus aureus,* and LPS (lipopolysaccharide) from *Escherichia coli*. Gene expression analysis showed that KLH and LTA up-regulated forkhead box J1 (FOXJ1) and integrin beta 4 (ITGβ4) in the DT40 cell line [70].

HD11 is a macrophagous chicken cell line derived from chicken bone marrow cells transformed by the avian leukemia virus [71]. HD11 was used to test FOS-inulin’s ability to support chicken macrophages in the elimination of *Salmonella enteritidis* [72]. HD11 was also used to study the effects of organic extracts of milk thistle, turmeric, reishi mushroom, and shiitake on the innate immunity and viability of cancer cells. RT-qPCR was used to quantify interferon-α, IL1β, 6, 12, 15, 18, and the tumor necrosis factor superfamily 15 (TNFSF15). The culture of HD11 with the abovementioned plant extracts improved cell proliferation along with inhibiting the growth of cancer cells compared to control [28].

MQ-NCSU is a macrophage cell line derived from the spleen, used to test (and confirm) the immunomodulatory role of β 1-4 mannobiose [73]. Brisbin et al. (2015) tested the effects of three types of *Lactobacillus* bacteria on the immune system using MQ-NCSU [46].

LMH is not strictly an immune cell line because it contains poultry liver cells immortalized from hepatocellular carcinoma [74]. However, it can be used to study both the metabolic and immunomodulatory properties of bioactive compounds. For example, Spivey et al. (2014) tested the adhesion of *Lactobacillus* to epithelial cells using the LMH cell line. Such an experimental approach model predicted gastrointestinal colonization using an in vitro LMH model [54]. Intestinal epithelial cell lines are used to study responses to various bioactive compounds, such as probiotics [55,60]. The new cell line is used in various experiments, such as testing the effects of *Salmonella enterica*. This cell line is derived from chicken intestinal epithelium cells, and it is a clone of MM-CHiC 8E11 [75].

### 3.3. Ex Vivo Organ Cultures

Ex vivo organ cultures combine in vitro and in vivo studies, as this model is based on fragments of organs or tissues grown in vitro. As such, it mimics the natural reactions of organisms, but under controlled conditions. Ex vivo organ cultures may be used where the type of experiment is not capable of being performed on a living organism. Organoid cultures also allow the cultivation of a suitable in vitro model for different studies.

Table 4 describes the ex vivo organ cultures carried out in chickens. Most ex vivo organ cultures of chickens come from the ileum [50,55,60]. The digestive tract is a complex environment that maintains close contact with the host and substances from feed, microorganisms, parasites, and toxins [76]. The intestine is a barrier to external microorganisms and pathogens. It regulates the composition of the microflora inhabiting the intestines. The intestine is lined with a single layer of epithelial, endocrine, and immune cells [77]. Ghiselli et al. (2021) isolated and successfully cultured chicken intestinal epithelium cells, starting with intestinal cell aggregates [60].

Three-dimensional chicken enteroids derived from intestinal embryonic villi and adult crypts were obtained by Nash et al. [78]. These enteroids allow modeling infections with avian pathogens, such as *Salmonella typhimurium* and influenza A virus. In addition to enteroids made of epithelial cells, it is also possible to obtain intestinal organoids, which consist of both epithelial and mesenchymal cells [79,80,81]. One of the disadvantages of ex vivo organ cultures is that they are not always in agreement with in vivo data. Ex vivo cultures, although they mimic functioning organs, still lack certain gut characteristics [82], including physiological interactions with other parts of the body [83].

### 3.4. In Ovo Injections

In ovo injection can be another avian research model used to study the immunomodulatory effects of a wide range of bioactive compounds, but also many other substances such as vitamins, minerals, or amino acids. The advantages of in ovo injections include less stress for the injected embryos and less methodological effort, the promotion of early bowel maturation, and improved immune status [88]. It has been proven that in ovo injection of bioactive products and compounds also improves health, chicken development hatchability, and the body weight of the hatched chicks [89]. This method allows broiler chickens to stimulate the early immune response [90]. In recent years, the most popular compounds used in ovo are pro-, pre-, and synbiotics, as well as polysaccharides, such as β-glucan or antimicrobial peptides, which have antibacterial properties against various pathogens [91].

Various studies have shown that there are several possible injection sites: air cell, amniotic sac, directly into the chicken embryo, or yolk sac [92,93,94,95]. The preferred site for in ovo injection in the early stages of embryo development is the air cell, but for injections of vaccines and nutrients, the best time is around day 18 of egg incubation [88]. In every research study, in ovo injection site and time are crucial in maximizing eggs hatchability and chicks quality [96]. In commercial conditions, in ovo injection on day 18 of egg incubation is used in broiler chickens for vaccination, e.g., against Marek’s disease or in ovo feeding. Another convenient term dedicated to in ovo stimulation is the day 12 of egg incubation. The injection on the day 12 is influenced by a number of factors resulting from the structure of the embryo and fetal membranes at this stage of development. The chorio-amniotic membrane is fully developed and highly vascularized at this stage of embryonic development. As a result, substances that penetrate the membrane enter the blood vessel system and are further transported to the embryo. An additional argument is also the size and arrangement of the embryo in the egg, allowing the noninvasive introduction of the needle into the air chamber without risking puncture. The immunomodulatory properties of the in ovo administration of bioactive substances on day 12 of egg incubation have been extensively described by Siwek et al. (2018) [97].

### 3.5. In Vivo Models vs. In Vitro Models

There are many models of experimental animal studies, including in vivo (live animals) and in vitro (cell and tissue cultures) approaches [98]. In vivo studies are considered the most informative due to the level of complexity of the biological system. Conducting research on live animals allows one to assess the impact of a given experimental factor on production conditions, as well as analyze physiological processes occurring in the environment of the organism, and not in the cell population. On the other hand, they provide confounding factors that make it difficult to interpret the data, for example, in hormonal disorders caused by stress and discomfort [99]. Finally, experiments on live animals involve ethical issues. Laboratories are required to implement 3R strategies (reduction, refinement, and replacement of laboratory use of animals). One of the key elements of the 3R concept is the replacement of experimental animals with in vitro and ex vivo models [100].

Each model has its advantages and disadvantages. In vitro cultures are extremely useful and ethically acceptable, but they are only a simplified model. The ultimate objective of cell or tissue culture is to reflect in vivo conditions as closely as possible. In vitro models are usually used at initial research stages and are followed by experiments on living organisms (e.g., laboratory animals or clinical studies). The results obtained in vitro need to be validated in the living organism, at the presence of various confounding factors. Cell/tissue culture undergo various manipulations, which are not present in natural conditions, such as freezing or thawing cells. On the other hand, cell or tissue cultures allow for more repetitions in a shorter time, reduce interfering environmental factors, and are simpler. For example, one of the applications routinely performed on in vitro cultures is to evaluate the toxicity and efficacy of various compounds on cells [101]. The most basic response of cells is their viability/unprofitability in response to different concentrations of the test medium [102]. The widespread use of cell and tissue cultures is hampered by technical reasons and their instability [98]. Working with primary cells is much more complicated than working with cell lines. Primary cells need more time to grow than cell lines and have limited growth potential. Another aspect is that it is time-consuming to establish the optimal growth conditions for such cells. Cell or tissue cultures are quite costly in respect to the reagents and consumables. There is also need for the laboratory used for cell cultures to maintain sterile conditions, and to be equipped with incubators, microscopes, and laminar chambers.

### 3.6. Future Directions

Nowadays, many factors contribute to the growing interest in bioactive products in poultry farming. This is related to the growing awareness of animal welfare and the food quality and safety. A major reason for turning to natural compounds is the antimicrobial resistance developed by many microorganisms, including human pathogens. Therefore, the line of research revolving around bioactive products with immunomodulatory properties is very promising. The cell culture models that have been recently developed are definitely more complex, so that they can closely mirror the in vivo model. Such an approach facilitates the studying of the effects of the bioactive compounds and products, and the results can be more directly applied to improve the health status of animals. The directions for further development of this line of research is to search for natural alternatives to antibiotics and therapeutic compounds, e.g., against avian influenza [103].

## 4. Conclusions

Different types of bioactive products, such as herbs, spices, plant extracts, as well as prebiotics, probiotics, and synbiotics have been used as supplements in poultry due to their immunomodulatory effects. The development of new supplements that improve performance and health in poultry can be facilitated by the use of various biological models. There are several in vitro and ex vivo models that can be used to study the immunomodulatory role of bioactive compounds in poultry. Additionally, an in ovo model allows for the injecting of the tested products directly into the structures of the developing embryo. This article provides a detailed overview of the available protocols or models that can be applied in immune-related studies to assess the specific properties of bioactive compounds in poultry prior to animal studies.

## Figures and Tables

**Table 1:** Examples of bioactive products and compounds with immunomodulatory properties in chicken.

Bioactive Compound	Amount	Bird Models	Results	Reference
Arabinoxylan wheat bran (AXs)	Group A: AXs 100 mg/kg body weight/day Group B: AXs 200 mg/kg body weight/day Group C: AXs 300 mg/kg body weight/day	Industrial broiler chicks (Hubbard)	The results indicated a higher amount of anti-SRBC IgM in chickens in the experimental group compared to the control group. In the case of the amount of anti-SRBC IgG, it was also significantly higher in the experimental group than in the control group.	Adapted from Akhtar et al., 2012 [50]
Acemannan (ACM 1), a complex carbohydrate extracted from *Aloe vera*	500 μg ACM vaccinated intramuscularly (6 chickens) and seemingly vaccinated (6 chickens) 3 days and 9 days before experimental analysis	2-month-old White Leghorn chickens homozygous for the main histocompatibility haplotype B13	ACM 1 permanently and effectively increased the activation capacity of macrophages from the systemic immune compartment (especially from the blood and spleen after intramuscular injection) in chickens, especially for the production of NO.	Adapted from Djerba et al., 2000 [47]
Thyme oil extract	100 and 200 ppm (parts per milion) in the diet	1 day-old broiler chicks of mixed-sex Arbor-Acres	Thyme improved weight gain, feed intake, and feed conversion rate, improving the digestive system. Chickens fed thyme oil extract had lower cholesterol levels and higher red blood cells, packed cell volume, hemoglobin, and white blood cells.	Adapted from Al-Kassie. 2009 [44]
*Ficus religiosa*	I. Aqueous extract—100, 200, 300 mg/kg body weightII. Ethanol extract—100, 200, 300 mg/kg body weight	1-day broiler chicks–Cobb	Both types of extracts affected the immune system by improving cellular immune performance. The researchers also noted growth-promoting effects.	Adapted from Mumtaz et al., 2021 [45]
Combination of herbs fermented with probiotics: *Curcuma longa*, *Houttuynia cordata*, *Prunus mume*, and *Rubus coreanus*	Chickens in the experimental groups received the same feed containing 1% or 2% of a combination of fermented probiotics	20-day-old Ross broiler chicks from one healthy Salmonella-free parent herd	The combination of probiotic-fermented herbs increased immune activity in broiler chicks such as antibody production level in serum and increased survival against *Salomenlla gallinarum* in experimentally infected broiler chickens due to stimulation of a nonspecific immune response.	Adapted from Jung et al., 2010 [41]
*Sacharomyces boulardii* and *Bacillus subtilis*	1 × 10^6^ cfu/mL for 3-, 6- and 12-h *S. boulardii, B. subtilis* and coculture *S. boulardii* and *B. subtilis*, 1 μg/mL lipopolysaccharide, saline phosphate buffer added to the control group	Chinese crossed chickens—dendritic cells derived from chicken bone marrow	The treatment groups modulated the phenotype and biological functions of chi-BMDC. Upstream levels of MHC-II, CD40, CD80, and CD86 gene expression in the stimulated groups, toll-like receptors TLR1, TLR2, TLR4, and chicken-specific TLR15 expressions improved, and the accompanying factors myD88, TRAF6, TAB1, and NFk-B increased in all treatment groups compared to control. The NFk-B response was significantly higher in the treatment of LPS in all groups. In addition, IL-1β, IL-17, IL-4, TGF-β, and IL-10 contrast, the LPS groups showed a marked increase in IL-12, INF-γ, and IL-8 concentration levels compared to the control group.	Adapted from Rajput et al., 2014 [51]
*Lactobacillus acidophilus, Lactobacillus reuteri, Lactobacillus salivarius*	1 × 10^6^ CFU thermally killed *S. typhimurium*, live *L. acidophilus*, live *L. reuteri*, and live *L. salivarius*	22 commercial broiler chicks of mixed-sex at the age of 5 or 6 weeks. The mononuclear cells of the spleen and the tonsils of the spleen were isolated and cultured	The three lactobacilli induced a much higher expression of interleukin 1β in spleen cells than in cecal tonsil cells—more inflammatory response in the spleen than in the cecal tonsil cells. *L. acidophilus* was more effective in inducing T-helper-1 cytokines, while *L. salivarius* induced a more anti-inflammatory response.	Adapted from Brisbin et al., 2010 [42]
*E. faecium* AL41, *E. faecium* 31, *L. fermentum* AD1 and infected *Salmonella enterica* serovar *Enteritidis* (SE147)	200 μg *E. faecium* AL41, *E. faecium* H31, *L. fermentum* AD1 and Salmonella *enterica* infected serovar *Enteritidis* (SE147) 1 × 10^9^ cfu/ml	Healthy poultry reared under standard conditions—peripheral mononuclear blood cells (PMBC)	The results showed that *E. faecium* AL41 exhibited the highest immunostimulating effect on the expression of selected cytokines by PMBC from chickens after Salmonella infection.	Adapted from Husáková et al., 2015 [52]
Probiotic based on *Lactobacillus*	Probiotic added in the amount of 1 g/kg of feed	100-day broiler chicks Ross 308	The results indicate that probiotic bacteria influenced the local immune response characterized by altered subpopulations of gut intraepithelial lymphocytes and increased birds’ resistance to *E. acervulina*, reflecting reduced oocyst shedding.	Adapted from Dalloul et al., 2013 [53]

**Table 2:** Overview of primary bird cell cultures.

In Vitro Model	Cell Description	Tested Factor	Conditions of Maintaining and Stimulation	Results	References
Primary Cell Cultures					
Cecal tonsils mononuclear cells	Tissues (spleen and cecal tonsils) crushed on a 40-µm nylon cell strainer to obtain single-cell suspension and separated into mononuclear cells with Histopaque–1077	*Lactobacillus acidophilus*, *Lb. reuteri*, *Lb. salivarius*	41 °C and 5% moisturized CO_2_ incubator; stimulation for 3, 6, 12 and 18 h; PMI medium with 10% fetal bovine serum and 200 U/mL penicillin, 80 g/mL streptomycin, and 25 mg gentamicin	Probiotics such as live lactobacilli induced expression of IL-1β, IL-12p40, IFN-γ, IL-18, IL-10, and TGF-β.	Adapted from Brisbin et al., 2010 [42]
Mononuclear cells of the spleen	Unicellular suspensions were isolated from the spleen. Mononuclear cells were obtained using the Ficoll-Paque gradient	*L. paracasei*, *L. reuteri*, *L. brevis*, *L. plantarum*, *L. paracasei*, *L. murinus-animalis*, *L. buchneri*, *L. paracasei*	41 °C and 5% CO_2_; RPMI 1640 medium containing 10% chicken serum, 1% non-etheric amino acids, 1% L-glutamine, 1% streptomycin	Bacteria strains which had positive influence on in vitro proliferation of mononuclear cells of the spleen also had positive influence on specific humoral immune responses.	Adapted from Koenen et al., 2004 [65]
chBMDC	Chicken bone marrow dendritic cells	*Saccharomyces boulardii*, *Bacillus subtilis*	Maintained after obtaining cell culture for 6 days at 41 °C and 5% CO_2_; stimulation for 3.6 × 12 h; RPMI 1640 medium with 10% poultry serum, 1% nonessential amino acids, 1% L-glutamine, 1% streptomycin	*Saccharomyces boulardii* and *Bacillus subtilis* had an influence on TLR-mediated signaling to induce immunity in chBMDCs.	Adapted from Rajput et al., 2014 [51]
IEC	Intestinal epithelial cells	*Lactobacillus fermentum*, *Lb. rhamnosus*, *Lb. rhamnosus*, *Lb. plantarum*, *Lb. ramous*	37 °C, 5% CO_2_, and 95% humidity; DMEM supplemented with 5% fetal calf serum, 2 mmol/l L-glutamine, 20 ng/mL, mouse epidermal growth factor, 2 μg/mL insulin from the bovine pancreas, 100 U/mL penicillin-streptomycin	There were changes in AvBD9 expression between probiotic bacteria and various bacteria stimulation doses. The *Lactobacillus ramous* MLGA strain had the strongest potential to increase AvBD9 expression of all the lactobacillus strains studied.	Adatpted from Li et al., 2012 [59]
PBMC	Peripheral mononuclear blood cells. The culture came from blood taken from the wing vein of chicks	probiotic fermented combination of four herbs delivered against *Salmonella*	41 °C and 5% CO_2_; RPMI-1640 medium with 2% antibiotic-antimycotic.	The probiotic fermented combination of four herbs enhanced the immune activity in broiler chicken and increased the survivability against *Salmonella*.	Adapted from Jung et al., 2010 [41]
		*Enterococcus faecium*, *E. faecium*, *Lactobacillus fermentum*	39.5 °C and 5% CO_2_; RPMI 1640 medium with 10 mM and 10% FBS	Compared to other tested probiotics, *E. faecium* AL41 showed the highest immunostimulatory effect on the level of relative expression of selected cytokines and chemokines after *Salmonella* infection.	Adapted from Husáková et al., 2015 [52]
		LPS, CpG ODN (short, synthetic, single-stranded DNA molecules, containing unmethylated CpG motifs), Pam3CSK4 (bacterial lipoproteins), Zymosan (*S. cerevisiae*), GOS (galactooligosaccharides), *L. lactis* subsp. *cremoris*, *S. cerevisiae*	41.5–42.5 °C and 5% CO_2_; RPMI 1640 medium; 10% FBS, 1% GlutaMAX, 1% antibiotic-antimycotic (next phases cell culture without this substance); stimulation for 3, 6, and 9 h	*L. lactis* subsp. *cremoris* had immunostimulatory properties in chicken PBMC and were expressed by increased mRNA abundance of IL-1β, IL-6, IL-8, and IL-12p40.	Adapted from Slawinska et al., 2021 [56]

**Table 3:** Overview of immune-related avian cell lines and culturing conditions.

In Vitro Model	Cell Description	Tested Factor	Conditions of Maintenance and Stimulation	Results	References
Cell Lines					
DT40	Cell line B established from bursa lymphoma infected with avian retrovirus RAV-1	prebiotics: RFO, inulin, Bi2tos; probiotics: *Lactococcus lactis* subsp. *lactis*, *L. lactis* subsp. *cremoris*	Incubator CO_2_ 37 °C and 5%; stimulation for 9 h; 80% advanced RPMI 1640 medium, 20% fetal bovine serum with 1 mM sodium pyruvate, 2 mM L-glutamine, 4.5 g/L glucose, 100 U/mL penicillin, 100 μg/mL streptomycin and 50 μM mercaptoethanol	The combination of prebiotic inulin and probiotic *Lactococcus lactis* subsp. *lactis* SL2 provided the strongest regulation of genes associated with the immune system, which proves the immunostimulating potential of this synbiotic.	Adapted from Sławinska et al., 2016 [69]
		LPS, LTA, KLH antigens	Advanced RPMI 1640 medium with 20% fetal bovine serum and addition of sodium pyruvate, L-glutamine, glucose, penicillin, streptomycin, mercaptoethanol; 37 °C and 5% CO_2_; stimulation was carried out for 3, 6, 9, and 24 h	At 24 h after stimulation, KLH and LTA antigens significantly increased mRNA expression of the FOXJ1 and ITGB4 genes. LPS was not a powerful stimulator of the genes of interest.	Adapted from Dunislawska et al., 2017 [70]
HD11	macrophage-like chicken cell line	FOS-inulin	Cells grown overnight at 41 °C and 5% CO_2_; the cells starved in a serum-free medium for 2 h, stimulated for 5 h; RPMI 1640 medium with 8% thermally inactivated chicken serum and 4% thermally inactivated fetal bovine serum, antibiotics, glutamine, and nonessential amino acids	FOS-inulin has the ability to modulate the innate immune system, which shows increased *Salmonella* Enteritidis killing and decreased organ colonization by these bacteria.	Adapted from Babu et al., 2012 [72]
MQ-NCSU	macrophage-like cell line derived from spleen cells of Leghorn hens challenged with the strain of Marek’s disease virus	b 1-4 mannobiose (MNB)	41 °C and 5% CO_2_; RPMI 1640 medium with 8% FBS, 10% poultry serum, 5% tryptose phosphate broth and 50 g/mL penicillin-streptomycin, 5 × 10^−5^ M 2-mercaptoethanol	MNB’s ability to up-regulate the expression of genes involved in host defense and stimulate the formation of reactive oxygen and nitrogen species suggests that it can increase macrophages’ *Salmonella*-killing activity and may operate as a potent immunomodulator.	Adapted from Ibuki et al., 2011 [73]
LMH	epithelial cell line derived from hepatocellular carcinoma	Rimfampicin-Resistant *Lactobacillus* cultures (strains of *Lactobacillus crispatus*, *Lb. gallinarum*, *Bacillus subtilis*)	37 °C and 5% CO_2_; Waymouth’s MB 752/1 medium with 10% FBS	Variables other than adhesion, such as bile tolerance, have a role in lactobacilli persistence in the gastrointestinal tract of chicken.	Adapted from Spivey et al., 2014 [54]
CHIC-8E11 (MM-CHiC clone 8E11)	intestinal epithelial cells obtained from chicken	*Salmonella enterica*	37 °C and 5% CO_2_; Modified Eagle Dulbecco Medium (DMEM)/Ham’s F-12 with L-glutamine, penicillin-streptomycin, and bovine serum	This research showed that most isolates have similar infection phenotypes, and that isolates with different infection phenotypes can be used to find new genes or gene variations that influence epithelial infection, such as novel components involved in *Salmonella* adherence and the invasion of epithelial cells.	Adapted from Kolenda et al., 2021 [75]

**Table 4:** Overview of the chicken ex vivo organ cultures derived from the intestinal tissue and culturing conditions.

Tissue	Purpose	Tested Factor	Culture Dish, Medium and Duration	Results	References
Chicken duodenal loops	Study of the probiotic’s ability to bind AFB1	*Lactobacillus rhamnosus* LC-705, *Propionibacterium freudenreichii* subsp. *shermanii* JS	Falcon tube PBS, 1 to 30 min	The findings of this study clearly revealed that the probiotic mixture can bind AFB1 in vitro, slow down AFB1 absorption in the chick duodenum, and lower AFB1 levels in duodenal tissue ex vivo.	Adapted from Gratz et al., 2005 [84]
Chicken ileum	Investigate the properties of adherent joints in the intestines	Six LAB isolates (E1223, E3, E4, E5, E7, and E8) derived from spontaneously fermented maize	Falcon tube, PBS, 30 min	Antibiotic-resistant LAB isolates E5, E7, and E8 were able to stick to chicken ileal cells in vitro. E8 isolate performed better than E5 and E7 isolates. The isolates E5, E7, and E8 were 99% identical to the *Pediococcus pentosaceus* ATCC 25,745 strain.	Adapted from Hamida et al., 2015 [85]
Chicken ileum	Evaluation of the effect of hops β and lipopolysaccharide on cytokine gene expression	β-acid hops, lipopolysaccharide	Falcon tube, DMEM, 30 min	The study demonstrated the anti-inflammatory action of hops β-acids and, as a result, the potential immunomodulatory activity exerted on host tissue, since they were able to reduce the expression of proinflammatory cytokines even when an inflammatory producing substance was present (i.e., LPS).	Adapted from Bortoluzzi et al., 2016 [86]
Chicken ileum	Isolate and characterize lactic acid bacteria from poultry	Lactic acid bacteria of poultry origin isolated from the intestines of chickens and broilers	Falcon tube, RPMI 1640 supplemented with 1% fetal bovine serum 1 h	Six poultry LAB strains were found to have suitable in vitro probiotic properties.	Adapted from Reuben et al., 2019 [87]
Chicken intestinal epithelial cells	Isolation and culture	No factor to be tested, attempt to obtain and maintain IEC	24-well plates with Matrigel matrix, DMEM	The research proved that chicken intestinal epithelial cells can be isolated and maintained in culture, and that they can be used as an in vitro intestinal model for future research.	Adapted from Ghiselli et al., 2021 [60]
3D chicken enteroids from intestinal embryonic villi and adult crypts	Developing a model for host-pathogen interactions study	No factor to be tested, attempt to culture avian enteroids with multiple villus-crypt structures	Matrigel, Advanced DMEM/F12 supplemented with 10 mM HEPES, 2 mM L-Glutamine, 50 U/mL Penicillin/Streptomycin and 2% B27 supplement	The authors developed a procedure for differentiating leukocyte-containing avian enteroids with an accessible epithelial layer. These enteroids mimic their in vivo counterparts’ 3D architecture, polarity, barrier function, and cellular composition, making them a good in vitro model of the post-hatch and mature chicken gut.	Adapted from Nash et al., 2021 [78]
Intestinal organoids	Establishment of 3D culture of intestinal organoids	No tested factor, protocol for 3D of epithelial organoids	Matrigel; DMEM/Ham’s F12, GlutaMAX, antibiotic-antimicotic solution, insuin-transferrin-selenium reagent, human recombinant R-spondin 1, human recombinant Noggin, AEGF, PGE_2_	The method of intestinal organoid culture derived from intestinal tissue fragments extracted from 18- to 20-day-old chicken embryos and placed in Matrigel was effectively introduced by the authors.	Adapted from Pierzchalska et al., 2016 [79]
	Establishment of isolation and culture method for chicken small intestinal organoids	Various concentrations of growth factors in order to obtain optimal conditions for culture	Matrigel 3-D culture system; BASIC MEDIUM: DMEM/F12 culture medium (containing 10 mM HEPES, 100 U/mL penicillin, 100 mg/mL streptomycin, 20 mg/mL nystatin, and 2 mM glutamax, pH 7.4) and 3 groups containing various additives to the medium	This study showed that a culture medium containing 50 ng/mL EGF, 100 ng/mL Noggin, and 500 ng/mL R-spondin 1 may effectively enhance the growth of chicken intestine organoids in vitro.	Adapted from Li et al., 2018 [80]
